# Preparing computed tomography images for machine learning in forensic and virtual anthropology

**DOI:** 10.1016/j.fsisyn.2023.100319

**Published:** 2023-02-08

**Authors:** Martin Lo, Enrico Mariconti, Sherry Nakhaeizadeh, Ruth M. Morgan

**Affiliations:** a UCL Department of Security and Crime Science, University College London, 35 Tavistock Square, London, WC1H 9EZ, UK; b UCL Centre for the Forensic Sciences, University College London, 35 Tavistock Square, London, WC1H 9EZ, UK

## 1 Introduction

Understanding human anatomy is fundamental to research and education related to medicine and forensic anthropology [1,2]. Physical specimens such as cadaveric prosections, dissection or, human bone collections are arguably irreplaceable for anatomical teaching [3,4]. However, the use of physical specimens introduces significant ethical considerations [5,6], as well as limitations related to cost and a gradual diminishing of specimen quality due to time and prolonged handling. The advent of medical imaging and its different modalities have provided research fields related to anatomy with the means to examine and visualise internal morphologies such as bone and soft tissues without the need for dissections or serial sections [7,8]. With digitisation techniques becoming increasingly utilised in archaeological and anthropological sciences [9], computed tomography (CT) databases are being accessed as repositories of virtual skeletal data [10]. In forensic anthropology, relevant techniques in scanning and processing of CT images are utilised in the development of novel methods for identification [[11], [12], [13], [14], [15], [16], [17], [18]]. CT scanning is a non-destructive acquisition method traditionally used in medical applications. CT scanning follows the basic physics principles of x-rays [7]. As x-rays traverse through matter, the beam undergoes attenuation. This effect is the reduction of x-ray beam intensity as it is either absorbed or deflected by the tissue [19]. In CT, a series of x-ray images of the object cross section are captured to construct three-dimensional visualisations with volumetric information [7,[20], [21], [22]]. While accessibility of CT data (including associated software) have improved in recent years through the efforts of open-source data repositories [23] and methods [24], dependencies on manual thresholding and segmentations continue to limit the number of images and sample size of data that can feasibly be processed for research. This has meant that it has been difficult to undertake research on modern day populations at sufficient scale to address identification challenges at sufficient scale.

The following is a pre-processing pipeline that has been created to remove irrelevant and non-osseous structures (such as fat, lymph nodes, and skin) or noise (such as the sliding table, stents, and medical tubing apparatus) in a CT scan. It offers the possibility of processing large numbers of CT scans to create large datasets that have relevance for the assessment of human remains in the context of modern populations. The pipeline presents three distinct outputs, 2D images thresholded for the tissue(s) of interest, stereolithography (STL) files for quantitative mesh analysis and 3D printing, and 3D image files readily available for machine learning applications.

## 2 Background

The increase in the application of medical imaging and digital data on the analysis of human remains and anatomy in forensic anthropology (FA) is well-documented [16]. This growing trend, now commonly referred to as virtual anthropology, has contributed to the development of quantitative forensic anthropology methodologies [11,[25], [26], [27]]. Medical imaging, specifically CT scanning, is able to provide high contrast digital image data that allow the slightest differences in tissues to be detected [19]. Structures scanned through CT can be visualised as high-fidelity grayscale images. These images can be further reconstructed as three-dimensional models or thresholded into images with regions/tissues of interest highlighted, enabling the investigation of complex skeletal morphology [28]. The features of CT have aided researchers across disciplines in the interpretation of medical images [7,8,29]. CT images are also used in other established and emerging research fields related to medicine, forensic anthropology, virtual anthropology, and 3D forensic science [10,[30], [31], [32]]. The advances in medical imaging technologies alongside disciplines such as computer science, has provided new ways of generating 3D visualisations of human anatomy for study and research from CT scans [33]. CT scans, recorded as Digital Imaging and Communications in Medicine (DICOM) data, can be visualised as two-dimensional image files in axial, coronal, and sagittal views, and the tissues of interest can be differentiated and highlighted using the differences in attenuation [34]. Attenuation is the overall decrease in the intensity of the emitted x-ray beam as it passes through the object due to processes such as absorption and scattering [19]. As result of the attenuation effect, dense tissue/material will appear bright, while less dense tissue/material will appear dark [35]. Further, the DICOM images can be stacked and reconstructed as a three-dimensional volume rendering [28]. Through three-dimensional reconstructions and renderings using DICOM data from CT, size and shape information of the scanned object, or anatomy of interest can be accurately determined [28,31]. Comparative studies have further shown a high degree of comparability between dry bones and three-dimensional models rendered from CT scanning [[36], [37], [38]]. As a result of this accuracy and continual efforts to adapt traditional FA methods into virtual settings enabled by CT, CT scanning has seen increased use and in some institutes it has become part of routine procedure in medico-legal investigations [26,27,31,39].

The growth of virtual anthropology has resulted in an increasing demand for CT data [31,40] that is currently being met by CT databases created using modern populations from clinical scans or post-mortem scans [9,41]. The New Mexico Decedent Image Database (NMDID) and other open-source data archives (such as The Cancer Imaging Archive (TCIA)) are reflective of contemporary populations. The virtual nature of these databases not only provides research opportunities for digital preservation, clinical diagnostics, and post-mortem examinations, but also provides the opportunity for non-destructive analysis of contemporary skeletal structures [42]. Using these valuable sources of data can provide a better representation of modern populations in comparison to historical skeletal collections [9,43]. Further, collections of high-resolution post-mortem and clinical CT databases eliminates the need for bone preparation, the deterioration of physical samples over time, and allows for easy data sharing and distribution for rapid analysis [28,44]. The virtual nature of CT and its associated imaging modalities allows access where physical examination is impossible [45,46]. The virtual access also provides users with the opportunity to engage in transnational research and analysis, with virtual reconstructions being analysed between institutions in different geolocations [27].

The recent increase in the availability of data from medical imaging and interest in virtual anthropology has resulted in studies which conduct estimation methods based on morphological feature analysis on digital models [11,26,42,[47], [48], [49], [50]]. This is typically done through the conversion of DICOM data into a surface model, an STL (stereolithography) file. With the success of machine learning techniques in analysing nonmedical images [51], there is currently a rise in number of studies which utilise machine learning (ML) and artificial intelligence on biological profile estimations in FA [14,16,17,46,52,53,[53], [53], [54], [55], [56], [57], [58], [59], [60], [61], [62], [63], [64], [65]]. ML studies in medicine and FA have shown that 3D image files restructured from DICOM images such as Neuroimaging Informatics Technology Initiative (NIfTI), Wavefront Alias (OBJ), and STL is gaining interest and may be a valuable file format for convolutional neural networks in processing volumetric data [66].

The limitation to study sample sizes can be seen as a two-fold problem. Lack of data availability, and the feasibility of manually processing high quantities medical images to acceptable file formats and standards. While the former is being addressed by the emergence of databases such as the NMDID, TCIA, and the usage of diagnostic clinical CT scans from hospitals and medical facilities, the latter is often overlooked in research literature. Forensic science and virtual anthropology studies involving CT scans often overlook or underemphasize the time taken to process medical images for machine learning. As medical images used in virtual anthropology tend to be from post-mortem and/or clinical settings, the scanned specimen may include irrelevant and unnecessary biological and non-biological structures such as the sliding table (as part of the CT scanning apparatus), medical tubing, clothing, fat, skin, or lymph nodes. While noisy structures can be removed in medical imaging programs such as 3D Slicer [67] using guides and resources developed [24], it is arguably unfeasible and time-consuming to undergo these steps if hundreds, if not thousands of 3D models or individual scans were required for machine learning studies in FA or VA.

Given the current interest to utilise ML in Forensic Anthropology and Virtual Anthropology, large-scale CT databases are being turned to as a viable source of data. However, the manual steps involved in removing noise and unnecessary structures from great quantities of CT images remain a challenge for researchers and the tight timings of research project cycles. The purpose of this technical note is therefore, to introduce a pre-processing pipeline based in Python 3. Allowing users to adapt the Python scripts provided to handle and automatically process CT images to remove noise, and to generate three discrete file outputs relevant to 3D modelling, machine learning, and image analysis in forensic and virtual anthropology.

## 3 Pre-processing pipeline

A pre-processing pipeline was designed for application with Python 3.8 programming language to remove noise, generate thresholded CT images, 3D models in STL format, and 3D image files in NIfTI format. This pipeline has been developed to run on all operating systems compatible with Python 3.8.0 and the packages required in the pipeline. The packages utilised to construct this pipeline include pydicom, dicom2nifti, scipy, skimage, ITK, and VTK among others. The code and additional detail regarding dependencies is available for review and use at https://github.com/preprocessing_pipeline.

**Fig. 1:**
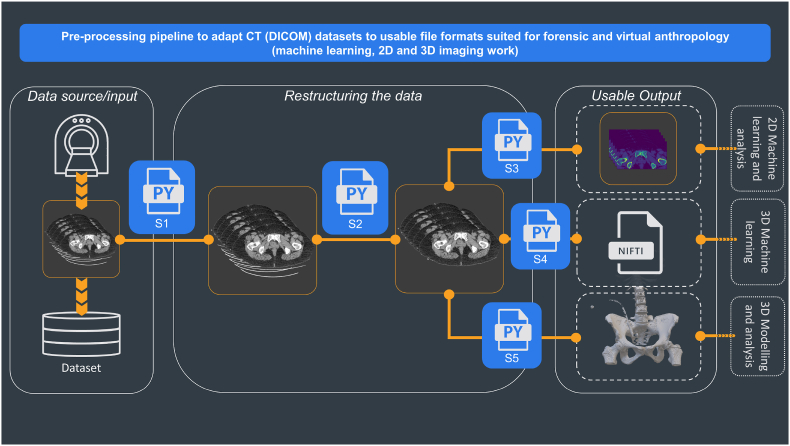
Pre-processing pipeline showing the steps for adapting CT (DICOM) datasets into file formats suitable for 2D and 3D image processing, virtual modelling, and machine learning work in forensic anthropology. The first stage is retrieving the data (Data Source), followed by the operations of data cleaning (Restructuring the data); the last stage is the Useable output, where different scripts prepare the scans for outputs required for the specifics of different types of research.

Fig. 1 provides a visual representation of the framework developed in this empirical section. The framework consists of three distinct stages, where each stage involves different Python scripts to produce the output desired.

(1) Data source/input
  (a) Dataset sorting and extraction script
(2) Restructuring data
  (a) Noise removal and segmentation script
(3) Useable output
  (a) Thresholding for tissues of interest script
  (b) Conversion to NIfTI format
  (c) Conversion to stereolithography format.

### 3.1 Data source/input

**Fig. 2:**
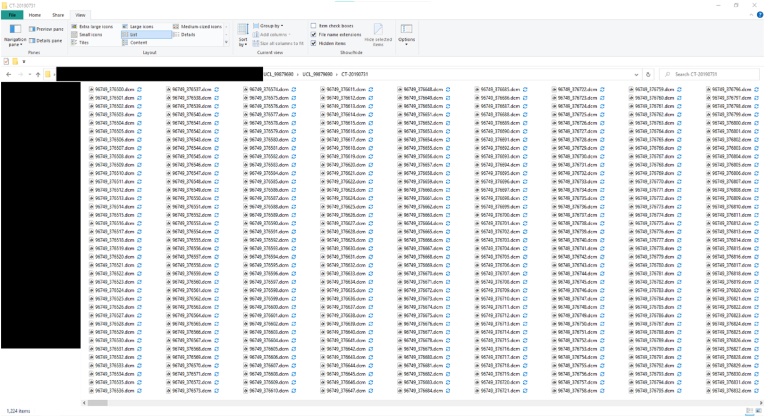
Uncollated DICOM files within individual CT study folder.

**Fig. 3:**
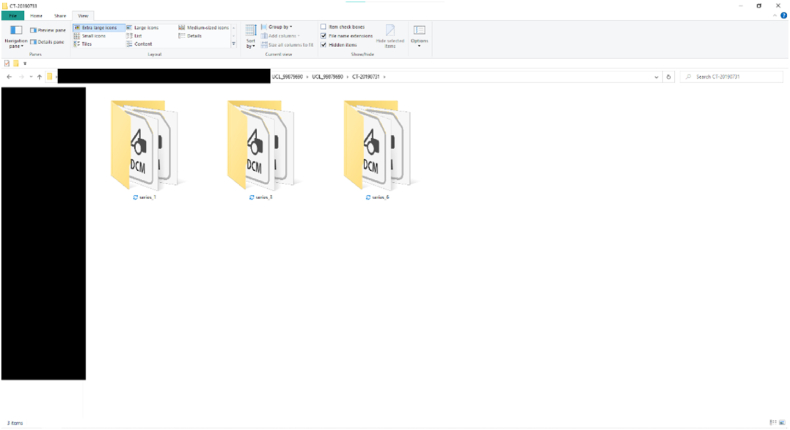
DICOM files automatically sorted and extracted into subfolders matching the series number in the metadata of each DICOM file.

The first set of scripts (S1) takes DICOM images as input and checks for corrupted and non-DICOM files, if found the identified files are deleted. It then sorts them into folders according to the series numbers. This enables the user to automatically sort and extract relevant DICOM files into folders created according to the series of each scan. Each CT study consists of multiple series of DICOM files; each DICOM file is an image consisting of an X-Ray slice of the body and the image metadata, while each series of DICOM files is the collection of the images of the scanned body. Each series may differ depending on the presence or absence of contrast agents or the scanned regions of interest. The metadata contain information such as slice thickness, series number, series date, patient sex, patient age etc. These fields or tags are defined by a pair of four alphanumeric characters [68], for example, (0020, 000E) denotes the *Series Number* tag of a DICOM file. While the DICOM files are generally extracted and provided in sequential order, each folder for a CT study for each patient could have multiple uncollated series of CT scans (Fig. 2). This first script therefore utilises the tags stored in each DICOM files' metadata with Pydicom [69], a python package capable of reading metadata to sort and extract DICOM files into its own ‘series folder’ within each CT study folder (Fig. 3). Finally, the third part of S1 enables users to delete DICOM files based on metadata information.

### 3.2 Restructuring the data

**Fig. 4:**
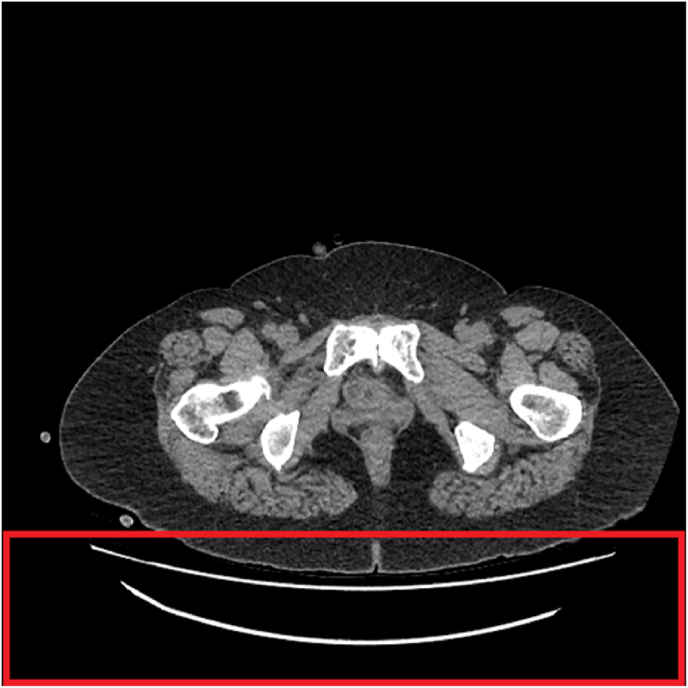
DICOM file with sliding table highlighted in red. (For interpretation of the references to colour in this figure legend, the reader is referred to the Web version of this article.)

The second script (S2) removes undesired structures such as the sliding table, which has been highlighted in Fig. 4. To ensure the script's adaptability for other CT scanning procedures, manufacturers, and protocols, the code in S2 was optimised so that a selection of the patient's tissues can be detected and the noisy structures outside of the selection can be removed. The selected section of the image matrix effectively becomes a mask; any sections outside of the selected mask is overwritten as ‘zero’, thus deleting the unwanted structures. As S2 directly overwrites the original file, metadata pertaining to each file is preserved. To achieve the described functions of S2, a few notable Python packages were used. The DICOM files were first thresholded using a combination of modules within the Pydicom package [69]. Thresholding highlights specific materials based on their radio density – measured in Hounsfield units [70]. The pixel matrix was thresholded to only display structures which had the radio density of bone tissue.

**Fig. 5:**
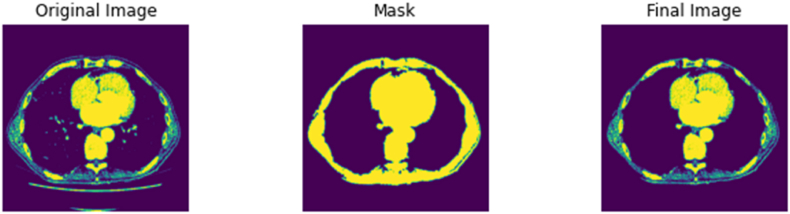
DICOM file labelled ‘Original Image’ undergoes a series of thresholding and masking functions to return an area of interest labelled as ‘Mask’. The ‘Final Image’ returned overwrites into the DICOM file, thus removing noisy structures such as the sliding gantry.

First, the DICOM file is loaded as an array of pixels and labelled as ‘Original Image’. This array of pixels undergoes a series of thresholding functions to only display the structures which has the radiodensity of bone and surrounding tissue. The thresholding values in S2 can be used as a fixed threshold if the tissue of interest is of bone tissue only. The selected area or area of interest is returned as a ‘Mask’, which denotes the parts of the pixel array that should be kept, returning an area of interest labelled as ‘Mask’. Using the area identified by the ‘Mask’, the ‘Original Image’ is segmented to produce the ‘Final Image’, the ‘Final Image’ array overwrites the original DICOM file while maintaining relevant metadata and the appropriate image matrix size (512 by 512) for future processing. The noise removal function of this script can be visualised in Fig. 5. In addition to the remove noise function above, S2 can detect corrupted DICOM files and non-DICOM files to be filtered and flagged for the user. This is to ensure the script's adaptability for other CT scanning procedures, manufacturers, protocols, and datasets from other sources. By removing incompatible and corrupted files, S2 enables the smooth transition to the next stage of the pre-processing pipeline.

### 3.3 Useable outputs

With the noise removed, the third script, S3, enables users to pass DICOM files through thresholding functions from Pydicom and coded to produce 2D general-purpose image formats. General-purpose image formats such as PNG (portable network graphics) and JPEG (joint photographic experts group) are regarded as standards and widely used as image storage formats and thus, commonly used in image-based machine learning [71]. As both PNG and JPEG are capable of compressed storage of image data without visible reductions of image quality, this conversion from DICOM to PNG/JPEG reduces the strain on computational power and resources [72,73]. Depending on the filetype requirements of the user/researcher, S3 can be altered to produce either PNG or JPEG files.

The fourth set of script (S4), enables users to compile and convert the processed DICOM files to the NIfTI file format using dicom2nifti package [74]. This package takes the collections of DICOM files sorted into each series folder to generate a single NIfTI file per series. By stacking the DICOM files, the resulting NIfTI file which contains volumetric data converted from the metadata and image data in the DICOM files is now fit for machine learning. Although originally created for neuroimaging, NIfTI is not limited to neurological tissue. Recent studies have demonstrated that NIfTI files can be thresholded through radiodensity thresholding to examine other soft tissues such as lung connective tissue and hard tissues such as bone [57,[75], [76], [77]]. With clinical and anatomical research demonstrating the feasibility of using NIfTI to examine bone tissues, this development implicates that the NIfTI format can be explored in the field of forensic and virtual anthropology. Moreover, the NIfTI format has been noted as the preferred format to conduct 3D machine learning as it contains the equivalent information of hundreds or even thousands of DICOM files in a single file [78]. Lastly, the NIfTI format is not exclusive of existing software, it is compatible with many current ready-made viewers, image analysis software such as 3DSlicer, ImageJ, InVesalius, OsiriX, R, and Python – through the Nibabel package [67,[79], [80], [81], [82], [83], [84]]. The second part of S4 enables users to move the generated NIfTI files into a new and separate directory.

The fifth script (S5), enables users to pass the processed DICOM files through the visualization toolkit (VTK) package, which is an open-source, Python compatible toolkit for 3D visualization and image processing [85], and contributes to the core of many open-source, multi-platform, ready-made software such as 3DSlicer, InVesalius, FreeSurfer [67,81,86]. The compiled DICOM files can be converted into stacks of array and thresholded to highlight the tissues of interest. The stacks of arrays utilise volumetric data from the DICOM files to form vertices and triangles which can be tessellated into a manipulable virtual model. The model can be written and saved as an STL file which can be visualised in virtual 3D viewing platforms/software or printed as a physical 3D model. The printed models provides tactile surface information of the scanned object [87,88]. This technology has since been incorporated into various fields of forensic science such as FA, crime scene reconstruction, ballistic reconstruction, and forensic medicine [[88], [89], [90], [91], [92]]. Recent research involving the usage of STL files have demonstrated that it can be used in both virtual and physical environments to an acceptable degree of accuracy and validity [11,93,94]. Through the automation potential presented by this script, large-scale datasets can be readily converted into STL files for either virtual manipulation or printed 3D structures for surface analysis or even used as demonstrative evidence in court [94,95]. Examples of the thresholded 2D image files, 3D models, and 3D image files produced by scripts S3 – 5 can be found in the examples folder of GitHub repository provided above.

## 4 Pipeline implementation

**Fig. 6:**
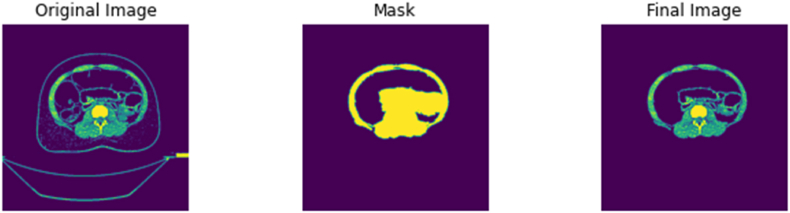
Noise removal and segmentation script on CT scan from the NMDID.

**Fig. 7:**
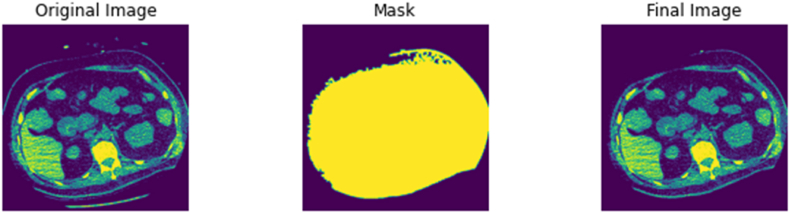
Noise removal and segmentation script on CT scan from the TCIA.

The pipeline described above was tested and implemented on a large-scale database of 3575 CT scans retrieved from the Picture Archiving and Communication System (PACS) office at University College London Hospital (UCLH). The scans were anonymised by removing all patient identifiers identified by NHS Patient Identification Policy and official documentation on Patient Safety Alert [96,97], leaving only minimal demographic information. Ethics approval was granted by the 10.13039/100005622Health Research Authority and 10.13039/100012068Health and Care Research Wales under protocol number 130244. The age of the patients ranged from 19 to 97 for females and 18–99 for males. All scans in the UCLH database were acquired using 120 kVp at 0.50 mm slice thickness on a single scanner – Aquillion ONE ViSION 320-detector row detector scanner (Toshiba Medical Systems, Otawara, Japan). Additionally, the pipeline was tested on single scans obtained from open-source public repositories NMDID and TCIA. The NMDID scans were acquired using 120 kVp at 0.50 mm slice thickness on the Philips Big Bore CT Scanner (Philips Medical Systems, Amsterdam, Netherlands), while the TCIA scans were acquired using 120 kVp at 0.625 mm slice thickness on an unknown CT scanner. Fig. 6, Fig. 7 show that the scripts, particularly the ‘noise removal and segmentation’ was able to remove the sliding table from CT scans from alternative datasets such as the NMDID and the TCIA as designed. S3 – 5 were also able to produce the discrete outputs as designed. Examples of the thresholded 2D image files, 3D models, and 3D image files from the single case files of the NMDID and TCIA databases can be found in the GitHub repository provided above.

## 5 Discussion

This pre-processing pipeline is an effective tool available to researchers that seeks to process large amounts of DICOM data from hospitals, forensic science institutes, scanned historical collections, or post-mortem databases to create any of the outputs offered for ML, 2D and 3D image analysis. This pipeline is flexible: the DICOM file standardisation allows the processing of any CT scans with little to no changes to the code provided. The processing thresholds and the noise removal (S2) have been tested and demonstrated against three different sets of scans from different data sources and regions of body anatomy. Consequently, the outputs provided at the end of the pipeline present high reliability outputs which can be used for multiple research purposes.

The capabilities shown by this preliminary pre-processing pipeline address both growing demands and problems faced by modern forensic and virtual anthropology. Firstly, the demand for modern population data is fully addressed by the pipeline as it has shown the capacity to transform clinical CT data and post-mortem CT data straightforwardly. Second, the pipeline addresses the limitations due to manual segmentations. While it has been noted by previously published studies that noise can be manually deleted through the use of visualization software programs [93], the task is time-consuming for larger data sets. This manual cleaning of noise ultimately impacts the sample size that a research study can feasibly process in the life cycle of the research project itself. As shown by the current ML research in FA, medical images have been shown to produce successful ML classification models which contribute to advanced ML techniques such as neural networks and random forests for age and sex estimation in post-mortem skeletal remains and living skeletal data. These studies utilise X-ray images [[98], [99], [100], [101], [102]], MRI images [103,104], photography [105], and CT scans [56,102,[106], [107], [108]] of bony anatomy to either cluster or classify individuals’ age and biological sex. While the growing body of literature has presented ML algorithms with promising results, study sample sizes are often limited to below 500 individuals/scans [17,64,65,109,110]. Though small datasets may be sufficient for the training of machine learning algorithms, the generalisability, accuracy, validity, and reliability of developed ML and AI models can be greatly improved with larger high-quality datasets [111].The larger sample sizes that the pipeline is able to produce will allow ML and AI models to decrease variability in predictive accuracies and increase model robusticity [112]. Additionally, the outputs of S2-4 are file formats are consistent with the inputs used in current ML research in FA. This pipeline therefore not only addresses the time-consuming problem removing undesirable background noise and structures, but enables robust future ML research in FA. The outputs produced by the pipeline can be used beyond ML and image analysis research. For example, as the pipeline is able to utilise large DICOM datasets to produce STL models via S5, it therefore serves as a tool to create virtual or printed 3D models representative of the contemporary population for training, teaching, and development purposes. Another example of pipeline flexibility can be seen in the output generated by S3, while the current script thresholds for bone tissue, and the thresholding value can be adjusted to highlight any tissue of interest. Thus, enabling the user to detect and highlight other tissues structures such as muscle. This flexibility allows the pipeline presented here to produce outputs that are relevant in fields outside of FA, e.g., medicine. Although this pipeline has shown success in processing different sets of CT data, it is noted that the STL models produced by S5 could be further improved. Surface differences were observed throughout the model, and it is noted that current step-by-step guides developed on open-source software will allow users to generate models of higher quality. While the scripts in the present pipeline were developed using CT scans, the code developed processes DICOM files. DICOM image files produced by other imaging modalities such as MRI or plain radiography can also be feasibly processed with some modification to the scripts.

The pipeline scripts are publicly available on GitHub for any interested stakeholders or researchers to download, use and validate to produce impactful work in relevant fields. This commitment to Open Science contributes to the transparency of the databases produced, and increases their value as tools in the evaluative interpretations made to assist the court [113,114]. Moreover, as this is the first tool published for the aforementioned purposes, the use of platforms such as GitHub allows the modification and improvement of the code by any interested member of the community. Therefore, we welcome any computationally advanced users of the pipeline to alter and refine the code provided, to generate higher quality models and other outputs on a mass scale. It is noted that the development of such a tool to process large quantities of medical image data could pose an impact to data privacy [115]. Medical images contain vast amounts of personal data in the form of the image and metadata that can be used to re-identify scanned individuals which violates privacy. However, it is noted in the published literature that medical images stored as DICOM images can be anonymised to reduce the risk of re-identification. Metadata associated with DICOM images is typically removed when these images are used for research purposes [116,117]. As the present pipeline uses DICOM images as the sole input format, future works that may utilise this pipeline would also adhere to the recommendations in the current literature to de-identify images. The potential of data privacy violations is mitigated with anonymisation processes and ethical safeguards in place.

## 6 Conclusion

The pipeline presented here is a set of novel procedures to prepare CT image data, specifically but not limited to clinical data, for forensic and virtual anthropology. The processes automated by the scripts found in the pipeline also allows a greater quantity of CT studies and scans to be processed, thus, removing the manual constraints such as manual segmentations and labelling that have limited the sample sizes of current research. With the automated processes in place, the pipeline grants the user with the flexibility to access and transform 10.13039/100004811CT datasets from virtually any source and of any size. Finally, the three distinct outputs provided by the pipeline enables the potentially large scale and automated production of 2D and 3D image files for machine learning purposes and 3D models for printing and virtual manipulation at a minimum. In the forensic anthropology context where funding, time, and access to modern population data may be restricted, this pipeline creates a process to transform 10.13039/100004811CT data from a variety of contexts such as post-mortem, living, or virtual preservations of historical collections into ad-hoc datasets with useable and relevant outputs.

## Disclosure statement

The authors declare no conflicts of interest were present in this research.

## Disclaimers

There was no external funding for this research.

## Presentation

This research was be presented at ANZFSS 2022 Brisbane, Forensics: Designing the Future. 11–15 Sept 2022 as a keynote presentation.

## Declaration of competing interest

The authors declare that they have no known competing financial interests or personal relationships that could have appeared to influence the work reported in this paper.
